# Anti-Pigmentary Natural Compounds and Their Mode of Action

**DOI:** 10.3390/ijms22126206

**Published:** 2021-06-08

**Authors:** Kyuri Kim, YoonJung Huh, Kyung-Min Lim

**Affiliations:** College of Pharmacy, Ewha Womans University, Seodaemungu, Seoul 03760, Korea; kyuri@ewhain.net (K.K.); huhlisa@naver.com (Y.H.)

**Keywords:** skin pigmentation, melanogenesis, *Tyrosinase*, natural compound

## Abstract

Hyper-activated melanocytes are the major cause of skin hyper-pigmentary disorders, such as freckles and melasma. Increasing efforts have been made to search for materials with depigmenting activity to develop functional cosmetics. As a result, numerous materials have been reported to have depigmenting activity but some of them are known to cause unwanted side effects. Consequently, anti-pigmentary natural compounds without concern of toxicity are in great demand. Virtually all sorts of natural sources have been investigated to find anti-pigmentary natural compounds. This review summarizes recently reported anti-pigmentary natural compounds and their mode of action from the ocean, plants, and bacteria.

## 1. Melanogenesis

Melanin plays a pivotal role in protecting the skin against UV radiation and oxidative stress from various pollutants, but its overproduction is the main cause of human skin hyperpigmentation [1]. Melanin is synthesized in the epidermal melanocytes by a process called melanogenesis. Each melanocyte in the basal layer of the skin epidermis is surrounded by approximately 36 keratinocytes [2]. During melanogenesis, melanin is stored inside the melanocytes in subcellular lysosome-like organelles called melanosomes, and transferred to the surrounding keratinocytes [3,4]. Melanogenesis happens with the activation of several melanogenesis-related proteins, including *Tyrosinase* (*TYR*), *Tyrosinase-related protein*-1 (*TRP-1*), and *Tyrosinase-related protein*-2 (*TRP-2*), under the orchestration of the principal melanogenesis regulating factor, *Microphthalmia-associated transcription factor* (*MITF*) [5].

There are two kinds of melanin pigment: brownish-black eumelanin and reddish-yellow pheomelanin [6]. The synthesis of melanin starts with the oxidation of L-tyrosine to *Dopaquinone* (*DQ*) and/or *L-dihydroxyphenylalanine* (*L-DOPA*) to *DQ* by *Tyrosinase* activation. *DQ* reacts with cysteine to form 5- or 3-cysteinyl DOPAs, which then oxidize and polymerize to ultimately make pheomelanin. Alternatively, *DQ* cyclizes to form a DOPA chrome, which is spontaneously decarboxylated to *5, 6-dihydroxyindole* (*DHI*). *DHI* rapidly oxidizes and polymerizes to yield *DHI*-melanin. In case *TRP-2* is available, DOPA chrome tautomerizes to form *DHI*-2-carboxylic acid (*DHI*CA), which can be oxidized and polymerized by *TRP-1* to yield *DHI*CA-melanin. The availability of substrates and enzymes decides the type(s) of melanin produced [3].

*MITF* is a crucial transcription factor stimulating the expression of *Tyrosinase*, *TRP-1*, and *TRP-2*. Multiple signaling pathways are involved in *MITF* regulation. UV exposure induces the activation of p53 and results in increased expression of proopiomelanocortin (POMC), which is then cleaved into small peptides including α-melanocyte-stimulating hormone (α-MSH). The POMC-derived α-MSH binds to the *Melanocortin-1 receptor* (*MC1R*) on the surface of melanocytes, leading to the production of cAMP through the activation of adenylyl cyclase. cAMP activates the *Protein kinase A* (*PKA*), which translocates to the nucleus and phosphorylates the cAMP response element-binding protein (*CREB*), causing the upregulation of *MITF* [7,8].

Additionally, *MITF* is controlled by the *Mitogen-activated protein kinases* (*MAPKs*), which are composed of three subtypes: *Extracellularly responsive kinase* (*ERK*), *c-Jun N-terminal kinase* (*JNK*), and p38 [9]. The phosphorylation of p38 activates the expression of *MITF* and eventually stimulates melanogenesis. On the contrary, *JNK* or *ERK* activation induces *MITF* degradation and downregulates melanin synthesis [8]. *MITF* is also activated by the Wnt/β-catenin pathway. Wnt is a cysteine-rich glycoprotein that couples with the G protein-coupled receptor to inhibit the glycogen synthase kinase-3β (GSK-3β). It causes the accumulation of β-catenin in the cytosol, increasing the upregulation of *MITF* [7].

When melanin production is completed, melanin contained in the melanosome is transferred to keratinocyte through melanocyte dendrites. Transfer of melanin is an important process in skin pigmentation, although much about the process is yet to be understood [10].

## 2. Skin-Whitening Agents Derived from Plants

Efforts to discover new plant extracts with skin-whitening efficacy have been made using various kinds of melanoma cells. In vivo evaluations using animal models such as mice or zebrafish have also been performed to confirm skin-whitening efficacy. Various parts of ginseng including the roots, seeds, and fruit have been widely used in both food and pharmaceutical products in East Asia for over 1000 years. Recently, a lot of research has been conducted to identify the skin-whitening ingredient from ginseng. Korean Red Ginseng (KRG) extract was verified as possessing inhibitory effects on melanin production in B16F10 cells through the suppression of *Tyrosinase* activity [11].

*Panax ginseng* calyx is the peduncle between the berry and root of ginseng. This study showed *Panax ginseng* calyx ethanol extract (Pg-C-EE) suppressed TRP1, TRP-2, *Tyrosinase*, *MITF*, p38, *ERK*, and *CREB* levels dose-dependently in B16F10 [12]. Ethanol extract of *Panax ginseng* seed was non-cytotoxic to Melan-A cells at a concentration of 100 ppm, at which it significantly (*p* < 0.01) suppressed cellular melanin production by inhibiting *Tyrosinase* activity [13]. Floralginsenoside A (FGA), ginsenoside Rd (GRD), and ginsenoside Re (GRE) purified from the *P. ginseng* berry also inhibited melanin production and *MITF* protein expression in melan-A cells [14]. Additionally, ginsenoside F1 (GF1), the major active component of *P. ginseng*, inhibited the melanin transfer on MNT-1/HaCaT coculture system and three-dimensional (3-D) human skin equivalent [15].

The extract from *Aster spathulifolius* extract (ASE) inhibited melanin production at concentrations of 50, 100, and 200 μM in B16F10 cells. Western blot analysis revealed that ASE extract activated both MAPK/*ERK* and Akt/GSK3β phosphorylation, promoting the ubiquitin-dependent degradation of *MITF*. The whitening effect of ASE was also observed on C57BL/6J mice, in which hyper-pigmentation was induced by 3, 6, and 9 weeks of UV irradiation. A significant reduction in melanin content was identified in the epidermis at a dose of 140 mg/kg [16].

Sweroside was isolated from *Lonicera japonica* and, upon testing, inhibited melanogenesis in Melan-A cells at 300 μM without cytotoxicity through decreasing *Tyrosinase*, *TRP-1*, and *TRP-2* expression. It has been shown that this effect occurred in a dose-dependent manner by promoting the Akt and *ERK* signaling pathways. In addition, sweroside was proven to be effective in vivo by reducing melanin spots in zebrafish embryos [17]. Isoliquiritigenin (ISL) is the hydrolysis product from licorice root. ISL inhibited melanin production through the degradation of *MITF* by activating the *ERK* signaling pathways, decreasing the expression of *Tyrosinase*, *TRP-1*, and dopachrome tautomerase (DCT) in SK-MEL-2 cells. Furthermore, ISL inhibited the melanocyte dendricity and melanin transport in cocultured SK-MEL-2 cells and HaCaT by suppressing the expression of Rab27a and Cdc42 proteins [18]. The ethanol extract of *Pueraria thunbergiana* significantly inhibited melanogenesis in B16F10 cells via anti-*Tyrosinase* activity. Phosphorylation of GSK-3β was decreased by *P. thunbergiana* in a dose-dependent manner, but the amount of phospho-*ERK* had no change, suggesting that *Pueraria thunbergiana* inhibits *MITF* through the Akt/GSK-3β pathway [19]. Leaf and root extracts from *Juglans mandshurica* (1–10 μM) suppressed *Tyrosinase* and *MITF* in B16F10 cells and primary human epidermal melanocytes (PHEMs). The extract was proved to be non-toxic up to 10 μM. The mechanism of suppression was revealed as a result of markedly enhanced phospho-*ERK* [20].

The ethanol extracts of *Sophora flavescens* root inhibited the transfer of melanin to keratinocytes via regulating both RAC1 in SK-MEL-2 cells and PAR2 in HaCaT cells. RAC1 promotes dendrite and lamellipodium formation in melanocytes. PAR2 stimulates the phagocytosis rate of keratinocytes and increases melanin transfer. The levels of RAC1, PAR2, and *MITF* mRNAs were suppressed following the treatment with *S. flavescens* extract [21]. A 70% ethanol extract of *Morus alba* leaves has an anti-melanogenesis effect via inhibiting *Tyrosinase* activity in α-MSH-activated B16-F10 cells in a dose-dependent manner. It inhibited *MITF*, *Tyrosinase*, and *TRP-1* by downregulating *CREB* and p38 signaling pathways [5]. Studies on the whitening effects of black tea extract (BT) have been conducted in several melanoma cells. Black tea water extract (BT) suppressed *MITF* in Mel-Ab cells [22]. *Camellia oleifera* extract was shown to inhibit melanin synthesis in B16F10 by suppressing *Tyrosinase* and TRP-2 production [23]. *Camellia sinensis* water extracts also significantly (*p* < 0.001) decreased *Tyrosinase* protein expression in a concentration-dependent manner [24]. Eupafolin (0.1–10 μM) isolated from *Phyla nodiflora* reduced cellular melanin content and *Tyrosinase* activity in B16F10 cells and had no cytotoxic effects. The mechanism was associated with the downregulation of *CREB* and *MITF*. It also induced the phosphorylation of *ERK*1/2 and p38 MAPK [25]. The following is a table listing plant extracts with skin-whitening effects and their mechanisms (Table 1).

## 3. Skin-Whitening Agents Derived from Ocean

The ocean has enormous biodiversity, with more than 250,000 species listed and many other species yet to be discovered. Marine resources have the properties of photoprotective, anti-aging, antioxidant, and moisturizing activities, attracting great attention from the cosmetic industry [26]. Besides, numerous compounds derived from marine organisms have already been investigated as *Tyrosinase* inhibitors [27]. The following is a table listing marine microorganisms with skin-whitening effects and their mechanisms (Table 2). These compounds show skin-whitening effects by regulating one or more steps of melanogenesis.

The *Tyrosinase* inhibitory activity of four types of extract, *Endarachne binghamiae, Schizymenia dubyi, Ecklonia cava* (EC)*,* and *Sargassum silquastrum* (SS), was measured at two temperatures. *Schizymenia* *dabyi* (SD) and *Sargassum silquastrum* (SS) extracts showed 90.75% and 70.75% inhibitory activity at 20 °C, respectively. *Endarachne* *binghamiae* (EB) and *Ecklonia cava* (EC) extracts showed 81.26% and 73.22% inhibitory activity at 70 °C, respectively. To investigate the inhibitory activities in vivo model, a zebrafish embryo was used. *Ecklonia* *cava* (EC) and *Sargassum silquastrum* (SS) extracts showed 48% and 50% inhibitory effects, respectively [28]. Fucoidan extracted from marine brown seaweed inhibited *Tyrosinase* catalytic activity competitively. [29]. Five compounds derived from *Ecklonia* *stolonifera*, OKAMURA, phloroglucinol, eckstolonol, eckol, phlorofucofuroeckol, and eckol, were treated to mushroom *Tyrosinase*. The inhibitory kinetics showed that phloroglucinol and eckstolonol were competitive inhibitors, and the remaining three compounds were noncompetitive inhibitors [30]. Diphlorethohydroxycarmalol (DPHC) from *Ishige* *okamurae* showed a 78.73% inhibition against mushroom *Tyrosinase*. It binds non-competitively to the enzyme and changes the shape of the enzyme, which leads to inhibit the binding of substrate to the active site [31]. Fucofuroeckol-A from *Eisenia bicylis* is a noncompetitive inhibitor of *Tyrosinase* and blocks IBMX-induced melanin formation [32]. *Turbinaria* *conoides* is a brown alga that produces zinc oxide. Inhibition activities were measured on *Tyrosinases* from potato and L-*TYR*osine. The combination of crude extracts of *Turbinaria* *conoides and* zinc oxide nanoparticles as a comparator showed 75% and 56% inhibition activity [33]. Methanol extracts of *Sargassum plagyophyllum* and *Eucheuma cottonii* were treated with L-tyrosine substrate and *L-DOPA* substrate. IC50 values for L-tyrosine substrate (monophenolase) were 2691.478 μg/mL and 2195.206 μg/mL. IC50 values for *L-DOPA* substrate (diphenolase) were 2631.648 μg/mL and 1769.336 μg/mL, respectively. IC50 values for diphenolase are less than those for monophenolase, which means the extracts have more significant effects on dephenolase [34]. Bromophenol obtained from *Symphyocladia* *latiuscula* exerts a strong hydrogen bond interaction with Arg268 and Per404 of *Tyrosinase*. Bromophenol only binds to free enzymes and works as a competitive inhibitor. Furthermore, it significantly reduced *Tyrosinase* expression levels [35]. Phlorofucofuroeckol A and 2-O-(2, 4, 6-trihydroxy phenyl)-6, 6′-bieckol from *Ecklonia* *cava* are slow-binding inhibitors. Both compounds interacted with His85 and Asn260 on the *Tyrosinase* active site, but each had different binding mechanisms. The former slowly binds to the active site following a single step-binding mechanism. The latter binds to the enzyme and induces a new conformational state of enzyme that inhibits substrate to make a complex with *Tyrosinase* [36]. Octaphlorethol A(OPA) from *Ishige* *foliaceae* inhibited melanin synthesis and *Tyrosinase* activity through the *ERK* pathway-mediated suppression of *MITF*, *Tyrosinase*, *TRP-1*, and *TRP-2* in α-MSH-stimulated B16F10 cells [37].

The skin-whitening effects of a marine-derived mixture from sea squirt skin were investigated. The mixture included a fucoidan-rich extract of *Undaria pinnatifida* (UPEF), a phlorotannin-rich extract of *Ecklonia cava* (ECE), and glycosaminoglycans (GAGs). Each component alone inhibited mushroom *Tyrosinase* in α-MSH-stimulated B16F10 cells but was cytotoxic. However, the combination of three components showed a stronger whitening effect without cytotoxicity. A mixture of compounds in a ratio of 4:5:1 (UEG-451) showed 71.1% whitening activity with the highest cell viability. Additionally, a UEG-451 combination downregulated melanogenesis-related proteins, including *Tyrosinase*, *TRP-1*, and *TRP-2*, in α-MSH-stimulated B16F10 cell by regulating the expression of *MITF* [38]. *Stichopus japonicus* extracts were evaluated by measuring the inhibition of mushroom *Tyrosinase* and melanogenesis in B16F10 melanoma cells. The results indicated that the extracts markedly inhibited *Tyrosinase* activity and melanin synthesis. *MITF* expression was also suppressed by the phosphorylation of *ERK*, leading to reduced melanogenesis-related gene expression [39]. Among *Streptomyces* extracts, Trichostatin A (TSA) showed the strongest *Tyrosinase* inhibition activity. TSA is a mixed-type inhibitor that can bind to the enzyme with or without substrates being bound [40]. Jeju magma-seawater (JMS) was pumped up from a depth of 130 m below sea level, and was found to have significant anti-melanogenesis activity. Since 100% of JMS showed cytotoxicity due to osmotic shock, research was undertaken at 25% and 50% concentrations. JMS activated CaMKKβ (calcium/calmodulin-dependent protein kinase β), which then activated AMPK (5′ adenosine monophosphate-activated protein kinase). AMPK blocks α-MSH-induced *MAPKs* (mitogen-activated protein kinase) and *PKA* (*Protein kinase A*) signaling pathways, resulting in the inhibition of *Tyrosinase* activity, melanin secretion, and melanogenesis-related gene expression [41]. *Pandina boryana* extracts regulated the phosphorylation of *ERK* and inhibited the expression of melanogenesis-related protein via the MAPK pathway [42]. Investigation with *Sargassum siliquosum* extracts with mushroom *Tyrosinase* revealed that 125 μg/mL of the extracts show 73% inhibition activity. The extracts have a phenol group; thus, the hydroxyl group of phenol makes a hydrogen bond with the active site of *Tyrosinase* that interrupts substrate binding with the enzyme and causes a conformational change in the enzyme [43]. One of the *Sargassum serratifoliu* extracts, Sargaquinoic acid (SQA), was investigated with mouse melanoma cells. SQA reduced cAMP by hydrophobic interaction with the binding site between cAMP and *PKA*. Reduced cAMP causes *PKA* inactivation, leading to the inhibition of *CREB* and *MITF* signals. Besides, the downregulation of *MITF* led the inhibition of melanogenesis-related protein expressions including *TRP-1*, *TRP-2*, and *Tyrosinase*. Additionally, SQA inhibited *Tyrosinase* by *MITF* degradation through the phosphorylation of *ERK*1/2 [44]. Chitosan is natural marine product that inhibits the synthesis of melanogenesis-related proteins and melanosome transfer. Chitosan was evaluated for its melanosome transfer interruption with human melanocyte cells and human keratinocyte cells. The results verified that the inhibitory activity of chitosan in melanocyte-keratinocyte was mediated by PAR-2, which is related to melanosome-transfer from melanocyte to keratinocyte. Chitosan inhibited melanosome release from melanocyte and melanosome uptake by keratinocyte [45]. 4-(phenylsulfanyl) butan-2-one from *Cladiella australis* (coral) exhibited a skin-whitening effect in B16-F10 cells (in vivo). *MITF*, *Tyrosinase*, *TRP-1*, *TRP-2*, and Gp100 were significantly reduced by *Cladiella australis*-4-(phenylsulfanyl) butan-2-one compared to the control group. Since there were no cytotoxic, carcinogenic, or teratogenic properties, the research suggested that 4-phenylsulfanyl-butan-2-one could be a safe and effective skin-whitening agent [46].

## 4. Skin-Whitening Agents Derived from Bacteria or Fungi

5-hydroxy-2-hydroxymethyl-4-pyrone, also known as Kojic acid, is naturally derived from fungal metabolites produced by species of *Acetobacter, Aspergillus*, and *Penicillium* [47]. Kojic acid has been used in concentrations of 2–4% either alone or in combination with 2% hydroquinone, as reported by Monteiro et al. According to the study, 0.75% Kojic acid with 2.5% vitamin C was shown to be a highly effective topical hypopigmenting agent in the treatment of facial melasma. Besides, the combination of glycolic acid with 5% Kojic acid showed better efficacy (28%) compared to glycolic acid with 5% hydroquinone (21%) [48]. Furthermore, Kojic acid is effective in regulating melanogenesis by inhibiting the catecholase activity of *Tyrosinase* in a non-classical manner [49]. Thus, kojic acid is widely used in cosmetics for its excellent whitening effect. *Saccharomyces cerevisiae* extract had inhibitory effects on melanogenesis and melanosome transfer.

*Tyrosinase* activity was blocked by the extract without cytotoxicity in B16F10 cells. However, the extracts had no effects on the expression of the *MITF* protein, which means they may indirectly inhibit *Tyrosinase* activity, but through post-transcriptional control. In addition, they inhibited PAR-2, melanosome transfer-related protein, so that melanosome transportation would be suppressed [50]. The effects of *Bifidobacterium adolescentis* on melanin contents and *Tyrosinase* activity were measured. Melanin content of a B16F10 melanoma cell treated with 7.5% *B.adolescentis* was 55.1% (% of control), and that of the positive controls was 86.4% (kojic acid) and 70.7% (arbutin). *Tyrosinase* activity of mushroom *Tyrosinase* treated with 7.5% *B.adolescentis* was 19.1%, and that of kojic acid and arbutin was 93.6% and 89.4%, respectively. These results indicated that *B.adolescentis* has a potent anti-melanogenesis activity superior to that of the positive controls, kojic acid, and arbutin [51]. *Angelica dahurica* root (ADR) was fermented with four different bacteria: *Bifidobacterium bifidum, Bifidobacterium lactis, Lactobacillus acidophilus*, and *Lactobacillus brevis.* IC50 for the *Tyrosinase* inhibition of extracts of ADR fermented by these four bacteria was 0.57, 2.80, 0.07, and 0.52 mg/m. Among the four bacteria, *L. acidophilus*-fermented ADR showed the highest activity. The IC50 of non-fermented ADR extract was 8.20 mg/mL, meaning that *Tyrosinase* inhibition activity of ADR has increased after fermentation [52]. Another study investigated the bioactive properties of *Lactobacillus rhamnosus* spent culture supernatant (Lr-SCS) for cosmetic applications. The *Tyrosinase* inhibitory activity of 100% Lr-SCS was 71.3% compared to 43.6% and 83.6% of 2 mM and 10 mM kojic acid. In this study, the lactic acid in Lr-SCS appeared to suppress tyrosine activity in a dose-dependent manner [53]. Lipoteichoic acid (LTA) is one of the cell wall components isolated from *Lactobacillus plantarum* (pLTA). pLTA decreased melanin synthesis by 57.9% compared to the control group. While pLTA did not inhibit *Tyrosinase* directly, it decreased intracellular activity of *Tyrosinase* and suppressed *Tyrosinase-related proteins TRP-1* and *TRP-2*. Furthermore, pLTA induced the phosphorylation of *ERK* and AKT, leading to *MITF* degradation [54]. *Lactobacillus acidophilus* (AL) is a popular probiotic strain, and the study investigated the effects of tyndallized *lactobacillus acidophilus* on melanogenesis. AL significantly inhibited melanin secretion and intracellular melanin content in α-MSH-treated melanocytes. Specifically, in α-MSH activated melanoma cells, AL inhibited the phosphorylation of *PKA* and *CREB* and suppressed the expression of *Tyrosinase* and *MITF*. However, when AL was treated on mushroom *TYR*osine to investigate *Tyrosinase* inhibition activity, there was no observed effect. Instead, AL reduced the mRNA expression of *Tyrosinase*, *TRP-1*, and *TRP-2*. Therefore, AL does not inhibit *Tyrosinase* directly but it can modulate melanogenesis through the regulation of the cAMP signaling pathway [55]. The extract of *Rhodobacter sphaeroides* (lycogen) was treated to B16F10 melanoma cell to evaluate anti-melanogenesis activity in vitro. The relative melanin content of lycogen-treated cells was significantly lower than the control group. To identify the mode of action of lycogen, the expressions of *Tyrosinase* and *MITF* were examined. Lycogen increased the *ERK* signaling pathway, which degraded *MITF*, leading to an anti-melanogenesis effect [56].

The anti-*Tyrosinase* activity of three *Bifidobacterium bifidum*-fermented Chinese herb extracts—walnut, Moutan cortex radices, and asparagus root—was investigated. *B. bifidum*–fermented extracts showed higher *Tyrosinase* inhibition activity than nonfermented extracts, suggesting that fermentation improved some biochemical activities of the three herb extracts. As a result, the highest *Tyrosinase* inhibition activities of the three fermented extracts were 58.4% (walnut), 66.5% (moutan cortex), and 92.8% (asparagus root) [57]. Urolithin A (UA) and Urolithin B (UB) are major metabolites found in human plasma. They can be produced from ellagic acid by *Gordonivater urolithincaciens* and *Gordonivater pamelaeae*, the urolithin-producing bacteria. Cytotoxicity and *Tyrosinase* inhibitory effects of UA and UB were evaluated in B16F10 melanoma cells. Cell viability was not affected by up to 10 μM of concentration. The melanin content of B16F10 cells treated with UA10 μM was 56.5%, which is comparable to that of the positive control (kojic acid). The result showed that, while neither Urolithin A (UA) or Urolithin B (UB) affected the mRNA expression of *Tyrosinase*, both competitively inhibited the enzymatic activity of *Tyrosinase* [58]. *Tyrosinase* inhibition activity of nonfermented *Rhodiola rosea* and *Lonicera japonica* extracts was 10 mg/mL and 6.93 mg/mL, respectively. However, the *Tyrosinase* inhibition activity of *Alcaligenes piechaudii*-fermented extracts was observed to be 0.78 mg/mL and 4.07 mg/mL, suggesting that the whitening effects of fermented *R. rosea* increased about 12 times, and that of *L. japonica* increased 70% compared to nonfermented extracts [59]. *Lactobacillus helveticus* NS8-fermented milk (NS8-FS) was examined for hypopigmentation. By measuring the melanin content and *Tyrosinase* activities in B16F10 cells, the whitening activity of NS8-FS was investigated. The color of the B16F10 cells was lightened and melanin content was reduced by nearly 40%. *Tyrosinase* activity was inhibited, and the gene expression of *TYR*osine-related protein was also downregulated. Therefore, NS8-FS could be both the enzymatic inhibitor and the gene expression regulator of *Tyrosinase* [60]. The anti-melanogenic activity of *Magnolia officinalis* bark (MOB) extracts after *Aspergillus niger* fermentation was investigated. The highest anti-*Tyrosinase* activity was shown with 3 days fermentation, which increased from 52.8% (before fermentation) to 93.6% (after fermentation). Additionally, the melanin content of human epidermal melanocytes was reduced. Melanin content was almost completely depleted at 200 μg/mL of fermented extracts [61]. *Asparagus cochinchinensis* extract fermented with *Aspergillus oryzae* was evaluated for its effects on cell viability and *Tyrosinase* inhibition. Cytotoxicity effects on HEMs, HaCaT, and A375.S2 cells were not significant. Anti-*Tyrosinase* activity of both fermented and unfermented extracts was evaluated in mushroom *Tyrosinase* and human epidermal melanocytes. Anti-*Tyrosinase* activity of the fermented extract was significantly lower in HEMs than in mushroom *Tyrosinase*, indicating some discrepancy in *Tyrosinase* inhibitory activity between mushroom *Tyrosinase* and human melanocytes. Additionally, with the fermented extract, the inhibition of melanin production was higher than *Tyrosinase* inhibition activity, suggesting that the fermented extract may downregulate not only *Tyrosinase* but also the *Tyrosinase-related protein MITF*. In contrast, the unfermented extract directly inhibited *Tyrosinase* activity [47,49,62]. The following table is a table listing skin-whitening agents derived from bacteria or fungi (Table 3).

## 5. Conclusions

In this review, we investigated plant-, ocean-, and bacteria-derived natural compounds with whitening effects and their modes of action. Most plant-derived skin-whitening agents suppressed melanin production by modulating the expression of melanogenesis-related proteins, such as *MITF*, *Tyrosinase*, *TRP-1*, and *TRP-1*. A few plant-derived skin-whitening agents promoted *ERK* signaling pathways, resulting in accelerated *MITF* degradation and regulating the length of melanocyte dendrites, which may block melanin transportation. Ocean-derived agents mostly suppressed melanin production by inhibiting the enzymatic activity of *Tyrosinase*, either competitively or noncompetitively. Bacteria-derived skin-whitening agents suppressed melanin production by regulating *Tyrosinase* activity and melanogenic proteins. Interestingly, fermented extracts showed higher inhibitory activity than non-fermented extracts, which may be owed, at least in part, to the combined effects of plant- and bacteria-derived active ingredients. Of note, most natural skin-whitening agents exhibited lower toxicity than existing skin-whitening agents, including arbutin and kojic acid. Since melanogenesis is complex and a variety of factors and pathways are involved, further research on the whitening activity of natural compounds is needed. Furthermore, although the whitening activity of these natural compounds has been well established in vitro, it is difficult to ensure efficacy in humans, since there are many confounding factors for manifesting whitening effects in human skin [63,64]. Thus, further investigation of these natural compounds’ whitening activity in vivo, in artificial skin models, or in clinical trials would be required to apply them to whitening cosmetics [65].

## Figures and Tables

**Table 1:** List of skin-whitening agents derived from plants.

Name	Mode of Action
Korean Red Ginseng (KRG)	*Tyrosinase* inhibition activity
*Panax ginseng* calyx ethanol extract (Pg-C-EE)	Inhibition of melanin production by suppressing *MITF*, p38, *ERK*, and *CREB*
The seed of *Panax ginseng*	*Tyrosinase* inhibition activity
Floralginsenoside A (FGA), Ginsenoside Rd (GRD), Ginsenoside Re (GRE)	Inhibition of melanin production by suppressing *MITF*
Ginsenoside F1 (GF1)	Inhibition of melanosome transport
*Aster spathulifolius* extract (ASE)	*Tyrosinase*, *TRP-1* and *MITF* suppressions by activating MAPK/*ERK* pathway.
Sweroside (*Lonicera japonica*)	*TRP-1*, *TRP-2*, and *MITF* suppressions
Isoliquiritigenin (ISL), licorice root	Tryosinase and *TRP-1* suppressions; induction of melanin degradation; reduction in the number of dendrites and melanin transport
*Pueraria thunbergiana*	*Tyrosinase*, *TRP-1, MITF*, and GSK-3β suppressions
*Juglans mandshurica*	*Tyrosinase* and *MITF* suppressions
*Sophora flavescens*	Inhibition of melanin transport
*Morus alba*	*Tyrosinase*, *TRP-1*, and *MITF* suppressions by downregulating *CREB* and p38 signaling pathways
Black tea extract (BT)	*Tyrosinase*, *TRP-2*, and *MITF* suppressions
*Camellia oleifera*	*Tyrosinase* and *TRP-2* suppressions
*Camellia sinensis*	*Tyrosinase* suppression
Eupafolin, *Phyla nodiflora*	Inhibition of Akt and activation of phospho-*ERK* or p38 MAPK

**Table 2:** List of skin-whitening agents derived from ocean.

Name	Mode of Action
*Endarachne binghamiae* *Schizymenia dabyi* *Ecklonia cava* (EC) *Sargassum silquastrum* (SS)	*Tyrosinase* inhibition activity
Fucoidan	Competitive inhibition of *Tyrosinase* activity
*Ecklonia stolonifera* OKAMURA	Noncompetitive and competitive inhibition of *Tyrosinase* activity
Diphlorethohydroxycarmalol (DPHC)	Noncompetitive inhibition of *Tyrosinase* activity
Fucofuroeckol-A	Noncompetitive inhibition of *Tyrosinase* activity
*Turbinaria conoides*	*Tyrosinase* inhibition activity
*Sargassum plagyophyllum Eucheuma cottonii*	*Tyrosinase* inhibition activity; protection against UV-B radiation-induced cell damage
*Symphyocladia latiuscala*	*Tyrosinase* inhibition activity
Phlorofucofuroeckol A and 2-O-(2, 4, 6-trihydroxy phenyl)-6, 6′-bieckol from *Ecklonia cava*	*Tyrosinase* inhibition activity
Octaphlorethol A(OPA) from *Ishige foliaceae*	*Tyrosinase* inhibition activity
Combination of *Undaria pinnatifida* (UPEF)*, Ecklonia cava* (E), glycosaminoglycans (GAGs)	*Tyrosinase* inhibition activity; *MITF* suppression
*Stichopus japonicus*	*Tyrosinase* inhibition activity; *MITF* suppression
Trichostatin A (TSA) from *Stichopus japonicus*	*Tyrosinase* inhibition activity
JMS (Jeju magma-seawater)	Inhibition of *Tyrosinase* activity; melanin secretion; melanogenic gene expression
*Pandina boryana*	Regulated phosphorylation of *ERK*
*Sargassum siliquosum*	*Tyrosinase* inhibition activity; conformational change of the enzyme
Sargaquinoic acid (SQA) from *Sargassum serratifoliu*	*TYR*, *TRP-1*, and *TRP-2* suppression
Chitosan	Inhibition of melanosome release and transport
4-(phenylsulfanyl) butan-2-one from *Cladiella australis*	*MITF*, *TYR*, *TRP-1, TRP-2*, and Gp100 suppression

**Table 3:** List of skin-whitening agents derived from bacteria or fungi.

Name	Mode of Action
*Acetobacter, Aspergillus* and *Penicillium*	Inhibition of *Tyrosinase* activity and melanosome transfer
*Saccharomyces cerevisiae* yeast extract	Inhibition of *Tyrosinase* activity and melanosome transfer
*Bifidobacterium adolescentis*	Inhibition of *Tyrosinase* activity and melanin production
*Bifidobacterium bifidum*, *Bifidobacterium lactis*, *Lactobacillus acidophilus*, *Lactobacillus brevis*	Inhibition of *Tyrosinase* activity; fermented extracts showed higher anti-*TYR*osine activity than nonfermented extracts
*Lactobacillus rhamnosus*	Inhibition of *Tyrosinase* activity
Lipoteichoic Acid (LTA) from *Lactobacillus plantarum*	Inhibition of *Tyrosinase* activity; inhibition of melanogenesis-related enzymes
*Lactobacillus acidophilus* (AL)	Inhibition of the mRNA expression of melanogenesis-related genes
*Rhodobacter sphaeroides* (lycogen)	Inhibition of melanogenesis-related protein
*Bifidobacterium bifidum*-fermented Chinese herb extracts	Anti-*Tyrosinase* activity; fermented extracts showed higher anti-*TYR*osine activity than nonfermented extracts
Urolithin A (UA) and Urolithin B (UB) from urolithin-producing bacteria	Inhibition of *Tyrosinase* activity
Nonfermented *Rhodiola rosea* and *Lonicera japonica* extracts *Alcaligenes piechaudii*-Fermented extract	Inhibition of *Tyrosinase* activity; fermented extracts showed higher anti-*TYR*osine activity than nonfermented
*Lactobacillus helveticus* NS8-fermented milk (NS8-FS)	Inhibition of *Tyrosinase* activity; regulation of melanogenesis-related gene expression
*Magnolia officinalis* bark (MOB) extracts by *Aspergillus niger* fermentation	Inhibition of *Tyrosinase* activity
*Asparagus cochinchinensis* extract fermented with *Aspergillus oryzae*	Inhibition of *Tyrosinase* activity; regulation of melanogenesis-related protein; expression; non-fermented extract directly inhibited *Tyrosinase* activity

## Data Availability

Not applicable.

## Abbreviations

*TYR*	*Tyrosinase*
*TRP-1*	*Tyrosinase-related protein*-1
*TRP-2*	*Tyrosinase-related protein*-2
*MITF*	*Microphthalmia-associated transcription factor*
*DQ*	*Dopaquinone*
*L-DOPA*	*L-dihydroxyphenylalanine*
*DHI*	*5, 6-dihydroxyindole*
α-MSH	α-melanocyte stimulating hormone
*MC1R*	*Melanocortin-1 receptor*
*PKA*	*Protein kinase A*
*CREB*	*cAMP response element binding protein*
*MAPKs*	*Mitogen-activated protein kinases*
*ERK*	*Extracellularly responsive kinase*
*JNK*	*c-Jun N-terminal kinase*

## References

[1] Brenner M., Hearing V.J. (2008). The protective role of melanin against UV damage in human skin. Photochem. Photobiol..

[2] Van Den Bossche K., Naeyaert J.M., Lambert J.J.T. (2006). The quest for the mechanism of melanin transfer. Traffic.

[3] Pillaiyar T., Manickam M., Jung S.H. (2017). Recent development of signaling pathways inhibitors of melanogenesis. Cell Signal..

[4] D’Mello S.A., Finlay G.J., Baguley B.C., Askarian-Amiri M.E. (2016). Signaling Pathways in Melanogenesis. Int. J. Mol. Sci..

[5] Li H.X., Park J.U., Su X.D., Kim K.T., Kang J.S., Kim Y.R., Kim Y.H., Yang S.Y. (2018). Identification of Anti-Melanogenesis Constituents from *Morus alba* L. Leaves. Molecules.

[6] Yardman-Frank J.M., Fisher D.E.J.E.D. (2021). Skin pigmentation and its control: From ultraviolet radiation to stem cells. Exp. Dermatol..

[7] Kumari S., Tien Guan Thng S., Kumar Verma N., Gautam H.K. (2018). Melanogenesis Inhibitors. Acta Derm. Venereol..

[8] Pillaiyar T., Manickam M., Jung S.-H. (2017). Downregulation of melanogenesis: Drug discovery and therapeutic options. Drug Discov. Today.

[9] Ye Y., Chu J.-H., Wang H., Xu H., Chou G.-X., Leung A.K.-M., Fong W.-F., Yu Z.-L. (2010). Involvement of p38 MAPK signaling pathway in the anti-melanogenic effect of San-bai-tang, a Chinese herbal formula, in B16 cells. J. Ethnopharmacol..

[10] Gillbro J.M., Olsson M.J. (2011). The melanogenesis and mechanisms of skin-lightening agents—Existing and new approaches. Int. J. Cosmet. Sci..

[11] Saba E., Kim S.H., Lee Y.Y., Park C.K., Oh J.W., Kim T.H., Kim H.K., Roh S.S., Rhee M.H. (2020). Korean Red Ginseng extract ameliorates melanogenesis in humans and induces antiphotoaging effects in ultraviolet B-irradiated hairless mice. J. Ginseng Res..

[12] Lee J.O., Kim E., Kim J.H., Hong Y.H., Kim H.G., Jeong D., Kim J., Kim S.H., Park C., Seo D.B. (2018). Antimelanogenesis and skin-protective activities of Panax ginseng calyx ethanol extract. J. Ginseng Res..

[13] Lee Y., Kim K.T., Kim S.S., Hur J., Ha S.K., Cho C.W., Choi S.Y. (2014). Inhibitory effects of ginseng seed on melanin biosynthesis. Pharm. Mag..

[14] Lee D.Y., Lee J., Jeong Y.T., Byun G.H., Kim J.H. (2017). Melanogenesis inhibition activity of floralginsenoside A from Panax ginseng berry. J. Ginseng Res..

[15] Lee C.-S., Nam G., Bae I.-H., Park J. (2019). Whitening efficacy of ginsenoside F1 through inhibition of melanin transfer in cocultured human melanocytes–keratinocytes and three-dimensional human skin equivalent. J. Ginseng Res..

[16] Hwang G.Y., Choung S.Y. (2016). Anti-melanogenic effects of Aster spathulifolius extract in UVB-exposed C57 BL/6J mice and B16F10 melanoma cells through the regulation of MAPK/*ERK* and AKT/GSK 3β signalling. J. Pharm. Pharmacol..

[17] Jeong Y.T., Jeong S.C., Hwang J.S., Kim J.H. (2015). Modulation effects of sweroside isolated from the Lonicera japonica on melanin synthesis. Chem. Biol. Interact..

[18] Lv J., Fu Y., Cao Y., Jiang S., Yang Y., Song G., Yun C., Gao R. (2020). Isoliquiritigenin inhibits melanogenesis, melanocyte dendricity and melanosome transport by regulating *ERK*-mediated *MITF* degradation. Exp. Derm..

[19] Han E., Chang B., Kim D., Cho H., Kim S. (2015). Melanogenesis inhibitory effect of aerial part of Pueraria thunbergiana in vitro and in vivo. Arch. Dermatol. Res..

[20] Kim J.Y., Lee E.J., Ahn Y., Park S., Kim S.H., Oh S.H. (2019). A chemical compound from fruit extract of Juglans mandshurica inhibits melanogenesis through p-*ERK*-associated *MITF* degradation. Phytomedicine.

[21] Shin D.H., Cha Y.J., Joe G.J., Yang K.E., Jang I.-S., Kim B.H., Kim J.M. (2013). Whitening effect of Sophora flavescens extract. Pharm. Biol..

[22] Choi S.-Y., Kim Y.-C. (2011). Whitening effect of black tea water extract on brown Guinea pig skin. Toxicol. Res..

[23] Zhu W.F., Wang C.L., Ye F., Sun H.P., Ma C.Y., Liu W.Y., Feng F., Abe M., Akihisa T., Zhang J. (2018). Chemical constituents of the seed cake of Camellia oleifera and their antioxidant and antimelanogenic activities. Chem. Biodivers..

[24] Kim Y.C., Choi S.Y., Park E.Y. (2015). Anti-melanogenic effects of black, green, and white tea extracts on immortalized melanocytes. J. Vet. Sci..

[25] Ko H.-H., Chiang Y.-C., Tsai M.-H., Liang C.-J., Hsu L.-F., Li S.-Y., Wang M.-C., Yen F.-L., Lee C.-W. (2014). Eupafolin, a skin whitening flavonoid isolated from Phyla nodiflora, downregulated melanogenesis: Role of MAPK and Akt pathways. J. Ethnopharmacol..

[26] Guillerme J.-B., Couteau C., Coiffard L.J.C. (2017). Applications for marine resources in cosmetics. Cosmetics.

[27] Burger P., Landreau A., Azoulay S., Michel T., Fernandez X.J.C. (2016). Skin whitening cosmetics: Feedback and challenges in the development of natural skin lighteners. Cosmetics.

[28] Cha S.H., Ko S.C., Kim D., Jeon Y.J. (2011). Screening of marine algae for potential *Tyrosinase* inhibitor: Those inhibitors reduced *Tyrosinase* activity and melanin synthesis in zebrafish. J. Dermatol..

[29] Yu P., Sun H. (2014). Purification of a fucoidan from kelp polysaccharide and its inhibitory kinetics for *Tyrosinase*. Carbohydr. Polym..

[30] Kang H.S., Kim H.R., Byun D.S., Son B.W., Nam T.J., Choi J.S. (2004). *Tyrosinase* inhibitors isolated from the edible brown algaEcklonia stolonifera. Arch. Pharmacal. Res..

[31] Heo S.-J., Ko S.-C., Kang S.-M., Cha S.-H., Lee S.-H., Kang D.-H., Jung W.-K., Affan A., Oh C., Jeon Y.-J. (2010). Inhibitory effect of diphlorethohydroxycarmalol on melanogenesis and its protective effect against UV-B radiation-induced cell damage. Food Chem. Toxicol..

[32] Shim K.B., Yoon N.Y. (2018). Inhibitory effect of Fucofuroeckol-A from Eisenia bicyclis on *Tyrosinase* activity and melanin biosynthesis in murine melanoma B16F10 cells. Fish. Aquat. Sci..

[33] Raajshree K., Durairaj B. (2017). Evaluation of the anti*Tyrosinase* and antioxidant potential of zinc oxide nanoparticles synthesized from the brown seaweed-turbinaria conoides. Int. J. Appl. Pharm..

[34] Dolorosa M., Purwaningsih S., Anwar E., Hidayat T. (2019). Tyrosinase inhibitory activity of Sargassum plagyophyllum and Eucheuma cottonii methanol extracts. IOP Conference Series: Earth and Environmental Science, 2019.

[35] Paudel P., Wagle A., Seong S.H., Park H.J., Jung H.A., Choi J.S. (2019). A new *Tyrosinase* inhibitor from the red alga Symphyocladia latiuscula (Harvey) Yamada (Rhodomelaceae). Mar. Drugs.

[36] Kim J.H., Lee S., Park S., Park J.S., Kim Y.H., Yang S.Y. (2019). Slow-binding inhibition of *Tyrosinase* by Ecklonia cava phlorotannins. Mar. Drugs.

[37] Kim K.-N., Yang H.-M., Kang S.-M., Kim D., Ahn G., Jeon Y.-J. (2013). Octaphlorethol A isolated from Ishige foliacea inhibits α-MSH-stimulated induced melanogenesis via *ERK* pathway in B16F10 melanoma cells. Food Chem. Toxicol..

[38] Wang L., Cui Y.R., Yang H.-W., Lee H.G., Ko J.-Y., Jeon Y.-J. (2019). A mixture of seaweed extracts and glycosaminoglycans from sea squirts inhibits α-MSH-induced melanogenesis in B16F10 melanoma cells. Fish. Aquat. Sci..

[39] Ding Y., Jiratchayamaethasakul C., Kim J., Kim E.-A., Heo S.-J., Lee S.-H. (2020). Antioxidant and anti-melanogenic activities of ultrasonic extract from Stichopus japonicus. Asian Pac. J. Trop. Biomed..

[40] Georgousaki K., Tsafantakis N., Gumeni S., Gonzalez I., Mackenzie T.A., Reyes F., Lambert C., Trougakos I.P., Genilloud O., Fokialakis N. (2020). Screening for *Tyrosinase* inhibitors from actinomycetes; identification of trichostatin derivatives from Streptomyces sp. CA-129531 and scale up production in bioreactor. Bioorganic Med. Chem. Lett..

[41] Song M., Lee J., Kim Y.-J., Hoang D.H., Choe W., Kang I., Kim S.S., Ha J. (2020). Jeju Magma-Seawater Inhibits α-MSH-Induced Melanogenesis via CaMKKβ-AMPK Signaling Pathways in B16F10 Melanoma Cells. Mar. Drugs.

[42] Jayawardena T.U., Sanjeewa K.A., Kim H.-S., Lee H.G., Wang L., Lee D.-S., Jeon Y.-J. (2020). Padina boryana, a brown alga from the Maldives: Inhibition of α-MSH-stimulated melanogenesis via the activation of *ERK* in B16F10 cells. Fish. Aquat. Sci..

[43] Arguelles E., Sapin A.B. (2020). Bioactive properties of Sargassum siliquosum J. Agardh (Fucales, Ochrophyta) and its potential as source of skin-lightening active ingredient for cosmetic application. J. Appl. Pharm. Sci..

[44] Azam M.S., Kwon M., Choi J., Kim H.-R. (2018). Sargaquinoic acid ameliorates hyperpigmentation through cAMP and *ERK*-mediated downregulation of *MITF* in α-MSH-stimulated B16F10 cells. Biomed. Pharmacother..

[45] Chen H.W., Chou Y.S., Young T.H., Cheng N.C. (2020). Inhibition of melanin synthesis and melanosome transfer by chitosan biomaterials. J. Biomed. Mater. Res. Part B Appl. Biomater..

[46] Wu S.-Y.S., Wang H.-M.D., Wen Y.-S., Liu W., Li P.-H., Chiu C.-C., Chen P.-C., Huang C.-Y., Sheu J.-H., Wen Z.-H. (2015). 4-(Phenylsulfanyl) butan-2-one suppresses melanin synthesis and melanosome maturation in vitro and in vivo. Int. J. Mol. Sci..

[47] Berardesca E., Rigoni C., Cantù A., Cameli N., Tedeschi A., Italia D.D., Laureti T., Agolzer A., Alfaioli B., Belloli C. (2020). Effectiveness of a new cosmetic treatment for melasma. J. Cosmet. Dermatol..

[48] Monteiro R.C., Kishore B.N., Bhat R.M., Sukumar D., Martis J., Ganesh H.K. (2013). A comparative study of the efficacy of 4% hydroquinone vs. 0.75% kojic acid cream in the treatment of facial melasma. Indian J. Dermatol..

[49] Cabanes J., Chazarra S., Garcia-Carmona F. (1994). Kojic acid, a cosmetic skin whitening agent, is a slow-binding inhibitor of catecholase activity of *Tyrosinase*. J. Pharm. Pharmacol..

[50] Lee W.J., Rhee D.Y., Bang S.H., Kim S.Y., Won C.H., Lee M.W., Choi J.H., Chang S.E. (2015). The natural yeast extract isolated by ethanol precipitation inhibits melanin synthesis by modulating *Tyrosinase* activity and downregulating melanosome transfer. Biosci. Biotechnol. Biochem..

[51] Huang H.-C., Chang T.-M. (2012). Antioxidative properties and inhibitory effect of Bifidobacterium adolescentis on melanogenesis. World J. Microbiol. Biotechnol..

[52] Wang G.-H., Chen C.-Y., Tsai T.-H., Chen C.-K., Cheng C.-Y., Huang Y.-H., Hsieh M.-C., Chung Y.-C. (2017). Evaluation of *Tyrosinase* inhibitory and antioxidant activities of Angelica dahurica root extracts for four different probiotic bacteria fermentations. J. Biosci. Bioeng..

[53] Tsai C.-C., Chan C.-F., Huang W.-Y., Lin J.-S., Chan P., Liu H.-Y., Lin Y.-S. (2013). Applications of Lactobacillus rhamnosus spent culture supernatant in cosmetic antioxidation, whitening and moisture retention applications. Molecules.

[54] Kim H.R., Kim H., Jung B.J., You G.E., Jang S., Chung D.K. (2015). Lipoteichoic acid isolated from Lactobacillus plantarum inhibits melanogenesis in B16F10 mouse melanoma cells. Mol. Cells.

[55] Lim H.Y., Jeong D., Park S.H., Shin K.K., Hong Y.H., Kim E., Yu Y.-G., Kim T.-R., Kim H., Lee J. (2020). Antiwrinkle and Antimelanogenesis Effects of Tyndallized Lactobacillus acidophilus KCCM12625P. Int. J. Mol. Sci..

[56] Liu W.-S., Kuan Y.-D., Chiu K.-H., Wang W.-K., Chang F.-H., Liu C.-H., Lee C.-H. (2013). The extract of Rhodobacter sphaeroides inhibits melanogenesis through the MEK/*ERK* signaling pathway. Mar. Drugs.

[57] Wang G.-H., Chen C.-Y., Lin C.-P., Huang C.-L., Lin C.-H., Cheng C.-Y., Chung Y.-C. (2016). *Tyrosinase* inhibitory and antioxidant activities of three Bifidobacterium bifidum-fermented herb extracts. Ind. Crops Prod..

[58] Wang S.-T., Chang W.-C., Hsu C., Su N.-W. (2017). Antimelanogenic effect of urolithin A and urolithin B, the colonic metabolites of ellagic acid, in B16 melanoma cells. J. Agric. Food Chem..

[59] Chen Y.-S., Liou H.-C., Chan C.-F. (2013). *Tyrosinase* inhibitory effect and antioxidative activities of fermented and ethanol extracts of Rhodiola rosea and Lonicera japonica. Sci. World J..

[60] Rong J., Shan C., Liu S., Zheng H., Liu C., Liu M., Jin F., Wang L. (2017). Skin resistance to UVB-induced oxidative stress and hyperpigmentation by the topical use of Lactobacillus helveticus NS 8-fermented milk supernatant. J. Appl. Microbiol..

[61] Wu L., Chen C., Cheng C., Dai H., Ai Y., Lin C., Chung Y. (2018). Evaluation of *Tyrosinase* inhibitory, antioxidant, antimicrobial, and antiaging activities of Magnolia officinalis extracts after Aspergillus niger fermentation. BioMed Res. Int..

[62] Wang G.-H., Lin Y.-M., Kuo J.-T., Lin C.-P., Chang C.-F., Hsieh M.-C., Cheng C.-Y., Chung Y.-C. (2019). Comparison of biofunctional activity of Asparagus cochinchinensis (Lour.) Merr. Extract before and after fermentation with *Aspergillus oryzae*. J. Biosci. Bioeng..

[63] Qian W., Liu W., Zhu D., Cao Y., Tang A., Gong G., Su H. (2020). Natural skin-whitening compounds for the treatment of melanogenesis. Exp. Ther. Med..

[64] Kim M.K., Kim K.B., Lee J.Y., Kwack S.J., Kwon Y.C., Kang J.S., Kim H.S., Lee B.M. (2019). Risk Assessment of 5-Chloro-2-Methylisothiazol-3(2H)-One/2-Methylisothiazol-3(2H)-One (CMIT/MIT) Used as a Preservative in Cosmetics. Toxicol. Res..

[65] Yun Y.E., Jung Y.J., Choi Y.J., Choi J.S., Cho Y.W. (2018). Artificial skin models for animal-free testing. J. Pharm. Investig..

